# Bioinformatics Identification and Experimental Verification of Disulfidptosis-Related Genes in the Progression of Osteoarthritis

**DOI:** 10.3390/biomedicines12081840

**Published:** 2024-08-13

**Authors:** Siyang Cao, Yihao Wei, Yaohang Yue, Deli Wang, Ao Xiong, Jun Yang, Hui Zeng

**Affiliations:** 1 National & Local Joint Engineering Research Centre of Orthopaedic Biomaterials, Peking University Shenzhen Hospital, Shenzhen 518036, China; 2 Shenzhen Key Laboratory of Orthopaedic Diseases and Biomaterials Research, Peking University Shenzhen Hospital, Shenzhen 518036, China; 3 Department of Bone & Joint Surgery, Peking University Shenzhen Hospital, Shenzhen 518036, China; 4 Department of Radiology, Peking University Shenzhen Hospital, Shenzhen 518036, China

**Keywords:** disulfidptosis, osteoarthritis, machine learning, hub gene, single cell analysis

## Abstract

Background: Osteoarthritis (OA) is a disabling and highly prevalent condition affecting millions worldwide. Recently discovered, disulfidptosis represents a novel form of cell death induced by the excessive accumulation of cystine. Despite its significance, a systematic exploration of disulfidptosis-related genes (DRGs) in OA is lacking. Methods: This study utilized three OA-related datasets and DRGs. Differentially expressed (DE)-DRGs were derived by intersecting the differentially expressed genes (DEGs) from GSE114007 with DRGs. Feature genes underwent screening through three machine learning algorithms. High diagnostic value genes were identified using the receiver operating characteristic curve. Hub genes were confirmed through expression validation. These hub genes were then employed to construct a nomogram and conduct enrichment, immune, and correlation analyses. An additional validation of hub genes was performed through in vitro cell experiments. Results: *SLC3A2* and *PDLIM1* were designated as hub genes, displaying excellent diagnostic performance. *PDLIM1* exhibited low expression in early chondrocyte differentiation, rising significantly in the late stage, while *SLC3A2* showed high overall expression, declining in the late differentiation stage. Cellular experiments corroborated the correlation of *SLC3A2* and *PDLIM1* with chondrocyte inflammation. Conclusions: Two hub genes, *SLC3A2* and *PDLIM1*, were identified in relation to disulfidptosis, providing potential directions for diagnosing and treating OA.

## 1. Introduction

Osteoarthritis (OA) stands as the most prevalent form of arthritis [1], impacting approximately 7% of the global population [2]. As the aging population and obesity rates rise, the prevalence of OA is expected to increase [3], posing significant and unavoidable challenges for global healthcare and public health systems [4]. OA is predominantly seen as an ailment of the elderly, with over one-third of those aged 65 and above showing OA in at least one joint [5,6,7]. However, factors such as obesity, physical activity, genetics, occupation, previous injuries, and socioeconomic status can influence osteoarthritic changes in the joints in younger populations [7,8]. Given our limited understanding of the molecular mechanisms driving the onset and progression of OA, current OA treatments primarily prioritize long-term pain relief, functional recovery, and an early resumption of daily activities [9]. Arthroplasty is considered in advanced cases [10]. The identification of innovative biomarkers is crucial for pinpointing potential targets for therapeutic interventions in OA.

Recent research has revealed the prominent roles of inflammation and programmed cell death (PCD) in chondrocytes in the progression of OA [11,12,13]. Therefore, focusing on chondrocyte cell death and uncovering the underlying regulatory mechanisms would facilitate the development of more effective OA treatment strategies. In February of 2023, Liu et al. from the MD Anderson Cancer Center reported the discovery of a novel type of PCD known as disulfidptosis. In disulfidptosis, a high expression of solute carrier family 7 member 11 (*SCL7A11*) and intracellular cystine accumulation leads to disulfide stress in cells experiencing glucose starvation [14]. Since its initial discovery, scholars have unveiled that the key genes linked to disulfidoptosis exert an influence on various diseases, including bladder cancer [15], hepatocellular carcinoma [16], esophageal carcinoma [17], lung adenocarcinoma [18], Alzheimer’s disease [19], and disorders of bone metabolism [20], among others.

Previous extensive metabolome analyses, conducted on a sizable sample, have revealed a positive correlation between the severity of radiographic OA and elevated plasma cystine levels [21]. Considering that an excessive accumulation of cystine in cells leads to disulfide stress, which results in disulfidptosis [18], it is reasonable to hypothesize that disulfidptosis may significantly impact the progression of OA. Despite its potential importance in OA, disulfidptosis has not been reported to be linked to OA. Therefore, further investigation is required to address this gap. In this groundbreaking study, single-cell and transcriptome data were used for the first time to identify hub genes—solute carrier family 3 member 2 (*SLC3A2*) and PDZ and LIM domain protein 1 (*PDLIM1*)—associated with disulfidptosis in OA. We speculate that these genes may influence the progression of OA, offering a new perspective to better understand the underlying molecular mechanisms of OA pathogenesis. Moreover, in vitro experiments were performed to explore the potential regulatory mechanisms of *SLC3A2* and *PDLIM1*, with the aim of elucidating the interaction between disulfidptosis-related genes (DRGs) and chondrocytes under inflammatory conditions. Taken together, our findings may proffer valuable insights for future in-depth studies and could hold translational potential for the development of novel pharmaceuticals for OA.

## 2. Materials and Methods

### 2.1. Data Sources

The OA-related datasets, namely GSE104782, GSE114007, and GSE57218, were acquired from the Gene Expression Omnibus (GEO) database (https://www.ncbi.nlm.nih.gov/gds, accessed on 1 October 2023). The GSE104782 single-cell dataset comprised 34 samples of articular cartilage tissue from individuals with OA. GSE114007 included cartilage samples from 20 OA patients and 18 normal individuals, while GSE57218 encompassed cartilage samples from 7 normal individuals and 33 patients with OA. DRGs were sourced from prior studies [22].

### 2.2. Identification of Differentially Expressed DRGs (DE-DRGs)

The ‘DESeq2’ package was employed to ascertain differentially expressed genes (DEGs) within the GSE114007 dataset, comparing samples from individuals with OA to those classified as normal. The statistical criteria for significance were set at *p* < 0.05 and |log2FoldChange (FC)| > 0.5 [23]. Utilizing the ‘ggplot2’ [24] and ‘pheatmap’ [25] packages, volcano and heat maps were generated to visually represent the expression patterns of the identified DEGs. The DE-DRGs were obtained by intersecting the DEGs and DRGs via the ‘ggVennDiagram’ package.

### 2.3. Function Analysis

Gene Ontology (GO), Kyoto Encyclopedia of Genes and Genomes (KEGG), and Gene Set Enrichment Analysis (GSEA) were conducted using the ‘clusterProfiler’ package with a significance threshold set at *p*-adj < 0.05 [26].

### 2.4. Machine Learning

The ‘caret’ package was employed to build Random Forest (RF) [27], Treebag, and Elastic Net models for screening feature genes derived from DE-DRGs. Subsequently, the ‘DALEX’ package was utilized to generate cumulative residual distribution diagrams and box plot distribution diagrams. The assessment of variable importance in the models was conducted through the application of the variable_importance function [28].

### 2.5. Identification of Hub Genes and Nomogram Construction

Receiver Operating Characteristic (ROC) curves were generated using the ‘pROC’ package [29]. Within the context of DE-DRGs, genes demonstrating substantial diagnostic potential (Area Under the ROC Curve (AUC) of DE-DRGs exceeding 0.8) were identified in GSE104782 and subsequently validated in GSE57218. The expression levels of genes with high diagnostic value were examined using the Wilcoxon rank-sum test. This analysis was further corroborated through expression validation in both GSE104782 and GSE57218 datasets. Hub genes were designated based on an AUC > 0.8 and a discernible difference in expression levels. Subsequently, a nomogram model was constructed using the ‘rms’ package [30] with hub genes as key components. The accuracy of the model in predicting OA was assessed through calibration and ROC curves.

### 2.6. Analysis of Immune Cell Infiltration

The ‘Immunedeconv’ package employed the CIBERSORT algorithm to deconvolve RNA expression profile data in GSE114007, providing estimates of the abundance of 22 distinct immune cell types [31]. The Wilcoxon rank-sum test was utilized to scrutinize differences in immune cell abundance between OA and normal conditions. Spearman correlation analysis was applied to investigate the associations between hub genes and differentially abundant immune cells.

### 2.7. Single-Cell Analysis

In the analysis of GSE104782, we employed the CreateSeuratObject function from the ‘Seurat’ package to perform filtering on the single-cell sequencing data, specifying criteria such as min.features = 200, min.cells = 3500 < nFeature RNA < 6000, and percent.mt < 3%. Subsequently, the data were standardized using NormalizeData. The identification of highly variable genes was accomplished through the application of the FindVariableFeatures function, with screening parameters set as method = vst and nfeatures = 2000 [32].

The normalization of the previously identified highly variable genes was executed using the ScaleData function, followed by principal component analysis (PCA). Additionally, linear dimensionality reduction was carried out through the application of JackStraw and ScoreJackStraw, facilitating the sorting of the percentage of variance represented by each principal component. Subsequently, the tSNE algorithm was employed to conduct dimensionality reduction analysis on samples, leveraging the information from the first 17 principal components identified earlier.

Cell types were annotated using the ‘singleR’ package [33], and Cellchat was employed to assess cell–cell communication. From the pool of 2000 highly mutated genes, those exhibiting the most significant expression variations between cells were chosen as feature genes for quasi-time series analysis. Pseudo-time series analysis, aimed at constructing the cell trajectory, was performed using the ‘monocle’ package [34]. An enhanced SCENIC method was applied to identify regulators of hub genes from single-cell transcripts. Furthermore, transcription factors (TFs) in different cell subtypes were determined based on regulon activity score (RAS) and regulon specificity score (RSS) [35]. Hub gene–protein/compound interaction networks were established using the Stitch database, with a confidence level set to 0.4.

### 2.8. Chondrocyte Culture, Treatment, and Small Interfering RNA (siRNA) Transfection

Primary mouse chondrocytes were purchased from Procell Life Science & Technology (CP-M087, Wuhan, China) and cultured in complete Dulbecco’s Modified Eagle’s Medium: Nutrient Mixture F-12 (DMEM/F12, PM150310B, Procell, Wuhan, China) at 37 °C in a 5% CO_2_ atmosphere. The first three passages of cells were used for all experiments. Chondrocytes were trypsinized with 0.25% trypsin-EDTA (G4001, Servicebio, Wuhan, China) and replated. To simulate inflammatory conditions resembling OA, chondrocytes were exposed to 10 ng/mL Interleukin-1β (IL-1β, 211-11B, PeproTech, Rocky Hill, NJ, USA) for 24 h.

Chondrocytes were seeded in six-well plates at a density of 1 × 10^6^ cells per well for experimental procedures. siRNA transfection occurred when cell density reached approximately 70%. Transfection involved using siRNA molecules targeting mouse *SLC3A2*, *PDLIM1*, or negative control siRNA (GenePharma, Shanghai, China) with GP-transfect-Mate (G04009, GenePharma, Shanghai, China) following the manufacturer’s instructions. Supplementary Table S1 contains the specific sequences of siRNAs targeting *SLC3A2* and *PDLIM1*. In experiments assessing the effects of *SLC3A2* knockdown, chondrocytes were treated with IL-1β (10 ng/mL) 24 h post-transfection. To evaluate the effects of *PDLIM1* knockdown under inflammatory conditions, chondrocytes were treated with IL-1β (10 ng/mL) 24 h before transfection.

### 2.9. Quantitative Polymerase Chain Reaction (qPCR)

Total RNA extraction from cells employed an RNA kit (082001, Beibei Biotechnology, Zhengzhou, China). Subsequently, the RNA underwent reverse transcription into cDNA using reverse transcription reagents (RR047A, Takara, Kyoto, Japan). qPCR was performed with TB Green^®^ Premix DimerEraser™ (RR091A, Takara, Kyoto, Japan) on the LightCycler 480 qPCR System (Roche, Basel, Switzerland). GAPDH served as the reference gene for normalizing other genes. The primer sequences for qPCR were synthesized by Sangon (Shanghai, China) and are outlined in Supplementary Table S2.

### 2.10. Protein Extraction and Western Blot

Protein extraction utilized RIPA lysis buffer (P0013C, Beyotime, Shanghai, China), and the subsequent separation of proteins occurred through SDS–PAGE gels (WB1102, Biotides, Beijing, China). Following this, protein transfer onto polyvinylidene difluoride membranes (ISEQ00010, Millipore, MA, USA) was conducted. Protein blocking was achieved using 5% skim milk (P0216, Beyotime, Shanghai, China). Subsequent incubation with primary and secondary antibodies was followed by the generation of a chemiluminescent signal using the super-sensitive ECL chemiluminescent substrate (BL520B, Biosharp, Hefei, China).

In this investigation, primary antibodies included SLC3A2 (ABclonal, Wuhan, China, #A23839, 1:1000), PDLIM1 (ABclonal, Wuhan, China, #A19224, 1:1000), GAPDH (Cell Signaling Technology, Danvers, MA, USA, #5174S, 1:10000), MMP3 (FineTest, Wuhan, China, #FNab05244, 1:1000), ADAMTS5 (ABclonal, Wuhan, China, #A2836, 1:1000), SOX9 (FineTest, Wuhan, China, #FNab10948, 1:1000), COX-2 (FineTest, Wuhan, China, #FNab10407, 1:1000), and HRP Goat Anti-Rabbit IgG (H+L) (ABclonal, Wuhan, China, #AS014, 1:10000). Protein levels were quantified using Image J/Fiji 2.9.0 (National Institutes of Health, Bethesda, MD, USA).

### 2.11. Statistical Analysis

Data were collected and independently analyzed by two researchers, SYC and YHW. The bioinformatics data underwent processing and analysis using the R software (version 4.2.1) and GraphPad Prism 9.5 (GraphPad Software, San Diego, CA, USA) was employed for molecular biology experimental data analysis. Quantitative data were obtained from a minimum of three independent experiments. Pairwise comparisons used Student’s *t*-tests, and multi-group comparisons utilized one-way analysis of variance (ANOVA). Results were presented as mean ± standard deviation (SD). All reported *p*-values were two-tailed, with statistical significance defined as *p* < 0.05. “ns” indicated no statistical difference, while significance levels were denoted as follows: *, *p* < 0.05; **, *p* < 0.01; ***, *p* < 0.001; and ****, *p* < 0.0001.

## 3. Results

### 3.1. Identification of Six DE-DRGs

**Figure 1:**
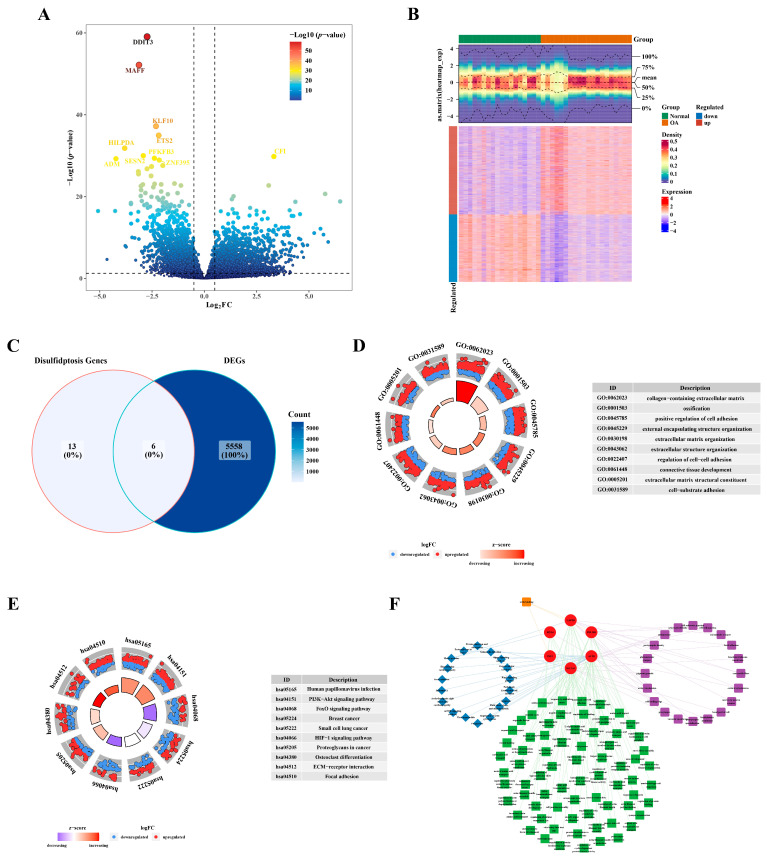
Identification of differentially expressed genes related to disulfidptosis. (**A**) Volcano plot illustrating the differential expression of genes. (**B**) Heatmap depicting the expression profile of differentially expressed genes. (**C**) Venn plot illustrating the intersection of identified genes. (**D**) GO enrichment analysis results. (**E**) KEGG enrichment analysis outcomes. (**F**) Network diagram visualizing the results of the enrichment analysis.

In GSE114007 (OA vs. Normal), a total of 5564 DEGs were identified, comprising 3153 up-regulated DEGs and 2411 down-regulated DEGs (Figure 1A,B). The intersection of DEGs and DRGs yielded six DE-DRGs: *SLC3A2*, *INF2*, *PDLIM1*, *MYL6*, *ACTB*, and *CAPZB* (Figure 1C). GO analysis revealed an enrichment of DEGs in processes such as the collagen-containing extracellular matrix, ossification, and the positive regulation of cell adhesion (Figure 1D). In the KEGG pathway analysis, the DEGs were implicated in human papillomavirus infection, the PI3K−Akt signaling pathway, and the FoxO signaling pathway, among others (Figure 1E). Utilizing the enriched terms from GO and KEGG, a network was constructed for DE-DRGs as illustrated in Figure 1F. For example, *PDLIM1* was enriched in postsynaptic density, the regulation of anatomical structure size, and the Rap1 signaling pathway. Furthermore, *CAPZB*, *PDLIM1*, and *INF2* exhibited enrichment in actin binding pathways.

### 3.2. Definition of Hub Genes: SLC3A2 and PDLIM1

**Figure 2:**
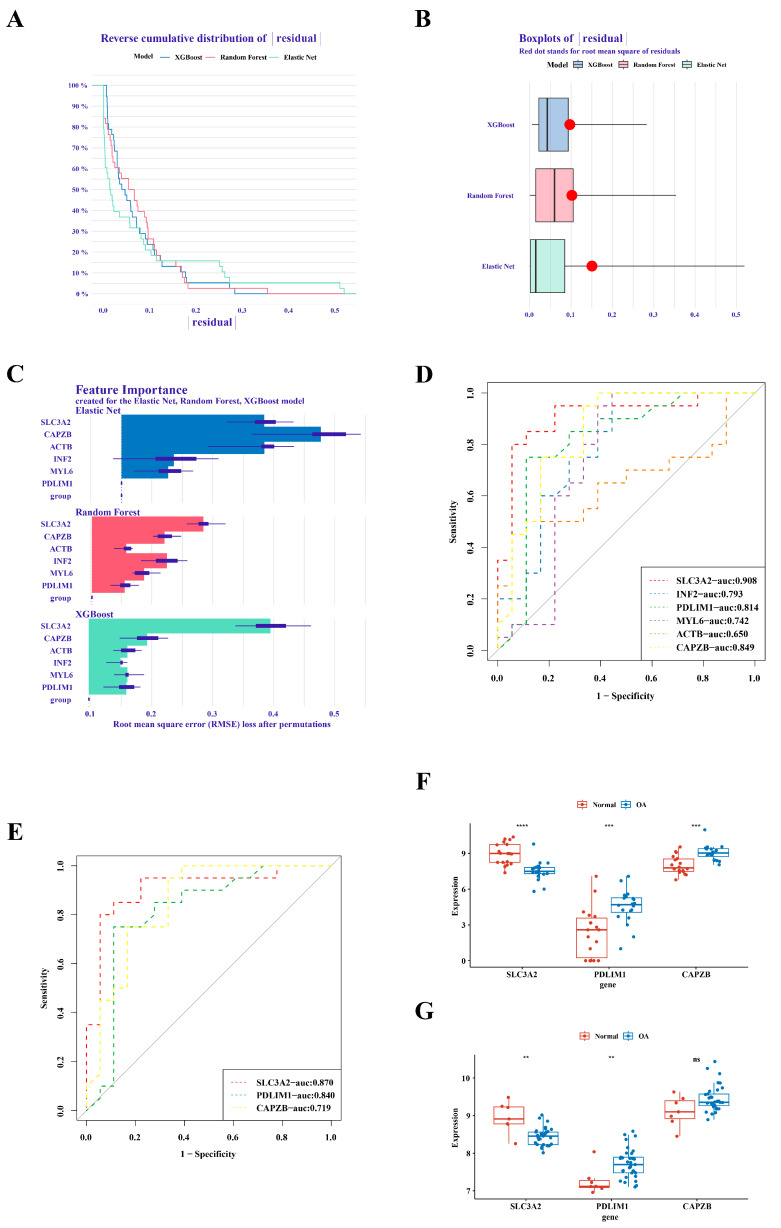
Identification of *SLC3A2* and *PDLIM1* as hub genes. (**A**) Cumulative residual distribution map illustrating model performance. (**B**) Boxplot of residuals demonstrating model performance metrics. (**C**) Importance analysis of model variables, emphasizing *SLC3A2* and *PDLIM1*. (**D**) Assessment of the diagnostic value of differential genes. (**E**) Verification of the diagnostic value of differential genes. (**F**) Expression validation of hub genes in the training set. (**G**) Validation of hub gene expression in the validation set. “ns” indicated no statistical difference, while significance levels were denoted as follows: **, *p* < 0.01; ***, *p* < 0.001; and ****, *p* < 0.0001.

In the analysis of GSE1140074, we employed three distinct machine learning methodologies, namely RandomForest, Treebag, and Elastic Net, for model construction. The comparative evaluation of model performance within the reverse cumulative distribution of residuals revealed a notable proximity among the outcomes of the three models (Figure 2A). An examination of residual boxplots elucidated that the Elastic Net model exhibited optimal performance, evidenced by the attainment of the smallest sample residual (Figure 2B). Further insight into feature importance delineated that *SLC3A2* and *CAPZB* held greater significance across all three models (Figure 2C). Within GSE1140074, the screening process identified three genes—*SLC3A2*, *PDLIM1*, and *CAPZB*—with high diagnostic value (AUC > 0.8), as depicted in Figure 2D. The validation of these findings in GSE57218 demonstrated that the AUC values for *SLC3A2* and *PDLIM1* remained above 0.8 (Figure 2E). Noteworthy distinctions in the expression trends of three genes in GSE1140074 and two genes (*SLC3A2* and *PDLIM1*) in GSE57218 between OA and normal samples were observed, while the expression of *CAPZB* exhibited no discernible difference in GSE57218 (Figure 2F,G). Through a comprehensive synthesis of ROC analysis and expression level verification, we conclusively designate *SLC3A2* and *PDLIM1* as pivotal hub genes in the context of our investigation.

### 3.3. Evaluation of the Nomogram Model’s Forecasting Accuracy

**Figure 3:**
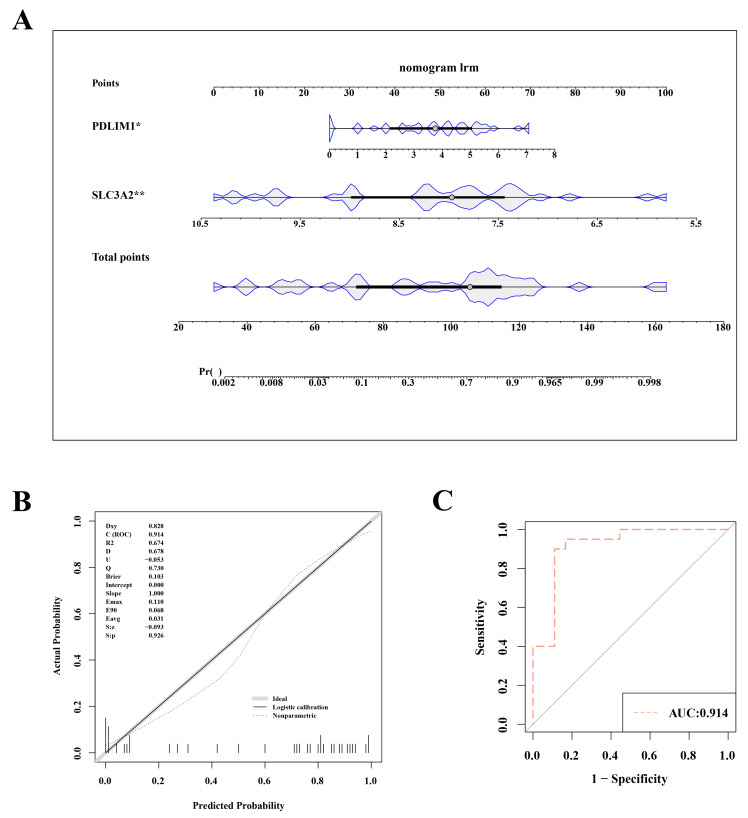
Nomogram model for accurate osteoarthritis risk prediction. (**A**) Nomogram depicting the risk estimation for osteoarthritis. (**B**) Calibration curve demonstrating the accuracy of the nomogram model. (**C**) ROC curve assessing the performance of the nomogram. Statistical significance was marked as *, *p* < 0.05; **, *p* < 0.01.

To assess and predict the risk of OA, we constructed a nomogram model based on the key genes *SLC3A2* and *PDLIM1*. This nomogram incorporated two hub genes (Figure 3A). The nomogram variables included the expression levels of *SLC3A2* and *PDLIM1*, with scores assigned based on their impact on OA risk. In both the training set (GSE114007) and the validation set (GSE57218), the nomogram model’s AUC exceeded 0.8, indicating high diagnostic value. The calibration curve slope and the AUC close to 1 suggest the model’s effective ability to distinguish between OA and normal samples (Figure 3B,C).

### 3.4. Single-Gene GSEA Analysis: SLC3A2 and PDLIM1

**Figure 4:**
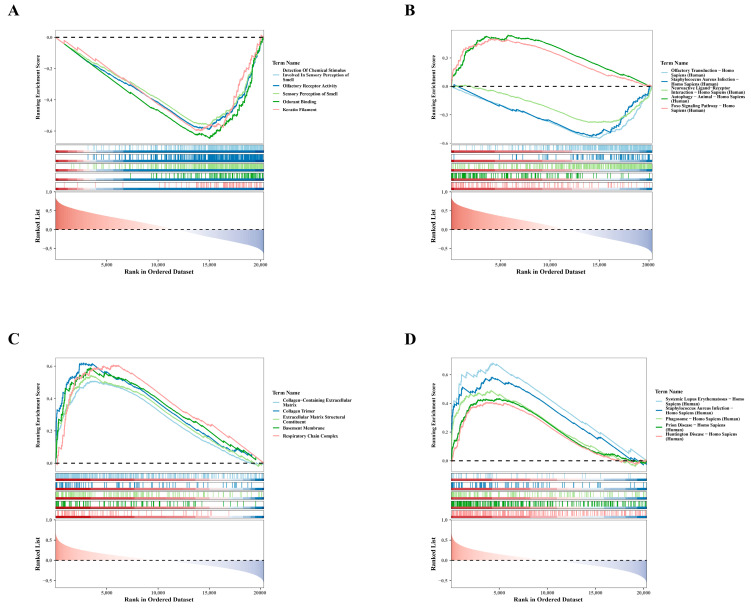
Single-gene GSEA analysis for *SLC3A2* and *PDLIM1*. (**A**) GO enrichment analysis revealing the functions of *SLC3A2*-associated genes. (**B**) KEGG enrichment analysis depicting pathways associated with genes related to *SLC3A2*. (**C**) GO enrichment analysis illustrating the functional annotations of *PDLIM1*-associated genes. (**D**) KEGG enrichment analysis showcasing pathways associated with genes related to *PDLIM1*.

To clarify the biological pathways associated with hub genes, we performed single-gene GSEA. For GO results, genes negatively correlated with *SLC3A2* were primarily enriched in processes related to the detection of chemical stimuli involved in olfaction, as shown in Figure 4A. The enrichment of these negatively correlated genes suggests potential regulatory mechanisms in olfactory function. Similarly, KEGG findings showed that genes positively associated with *SLC3A2* were mainly involved in autophagy in Homo sapiens, whereas genes negatively related to *SLC3A2* were involved in olfactory transduction in Homo sapiens (Figure 4B). This highlights the role of *SLC3A2* in regulating autophagy, which is crucial for chondrocyte health. Conversely, GO analyses showed that genes positively linked to *PDLIM1* were particularly associated with the collagen-containing extracellular matrix (Figure 4C). Meanwhile, KEGG results indicated that genes positively associated with *PDLIM1* were involved in systemic lupus erythematosus in Homo sapiens (Figure 4D), suggesting a role in inflammatory responses. These analyses revealed the crucial roles of *SLC3A2* and *PDLIM1* in OA, providing new insights into their potential biological functions.

### 3.5. Identification of Three Types of Differential Immune Cells

**Figure 5:**
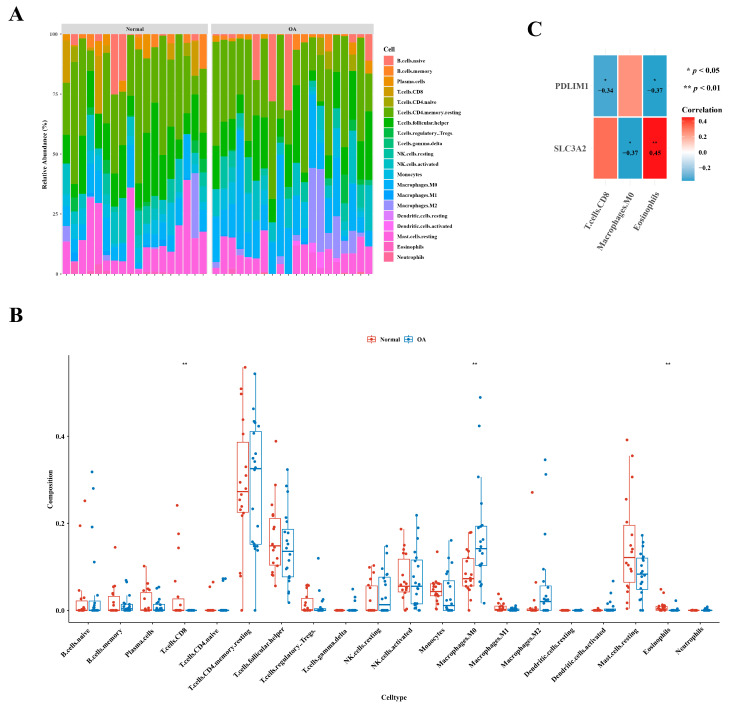
Distinctive immune cell types in osteoarthritis. (**A**) Comparative analysis depicting the relative abundances of immune cells in individuals with osteoarthritis in contrast to healthy controls. (**B**) Boxplot presenting the distribution of immune cell abundances specifically in osteoarthritis. (**C**) Exploration of gene relevance, emphasizing critical genes associated with immune cells.

Figure 5A illustrates the immune cell abundance in GSE1140074, comparing OA and normal samples. Distinct variations in the abundance of three immune cell types—specifically, CD8 T cells, M0 macrophages, and eosinophils—were evident between the two groups, as highlighted in Figure 5B. An examination of correlation results revealed a noteworthy positive correlation between eosinophils and *SLC3A2* (cor = 0.45), while the highest negative correlation was observed between M0 macrophages and *SLC3A2* (cor =−0.37), as depicted in Figure 5C.

### 3.6. Screening and Characterization of Ten Highly Variable Genes

**Figure 6:**
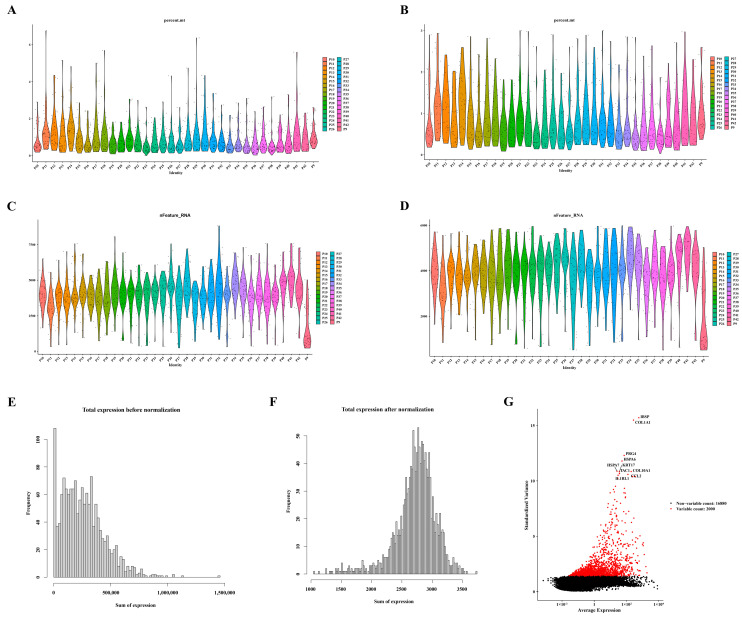
Identification of ten highly variable genes. (**A**) Violin plots displaying gene expression distribution before screening for mitochondrial genes. (**B**) Violin plots illustrating gene expression distribution after screening for mitochondrial genes. (**C**) Violin plots presenting gene expression distribution before screening for nfeature. (**D**) Violin plots depicting gene expression distribution after screening for nfeature. (**E**) Distribution of gene expression frequency pre-standardization. (**F**) Distribution of gene expression frequency post-standardization. (**G**) Selection process for highly variable genes.

Following the quality control process for the single-cell data from GSE104782, a refined dataset of 1471 cells was selected for subsequent analysis, as depicted in Figure 6A–D. The distribution of gene expression frequencies before and after standardization is presented in Figure 6E,F. From the extensive pool of genes, 2000 highly variable genes were meticulously identified. Notably, ten of these genes—namely *IBSP*, *COL1A1*, *PRG4*, *HSPA6*, *HSPA7*, *KRT17*, *COL10A1*, *CCL2*, *TAC*, and *ILRL1*—were specifically highlighted, as illustrated in Figure 6G.

### 3.7. Annotation of Six Cell Types from Twelve Cell Clusters

**Figure 7:**
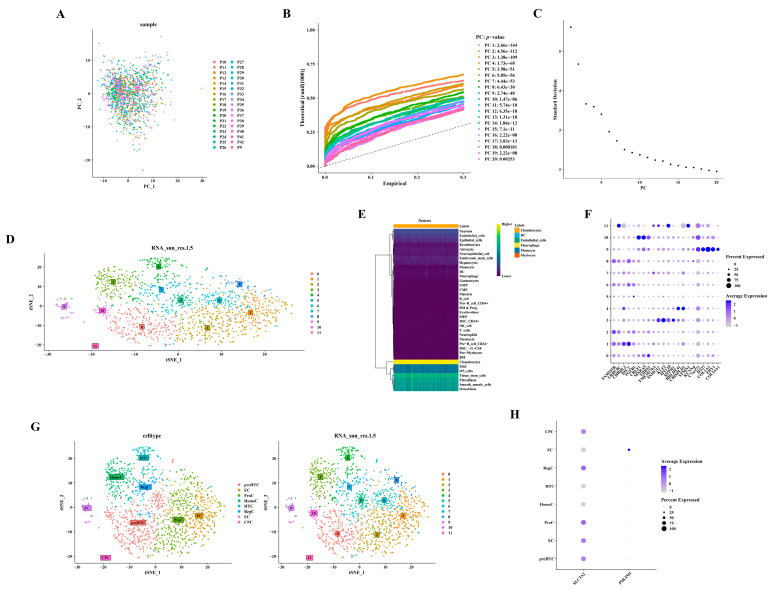
Annotation of six distinct cell types from 12 cell clusters. (**A**) Scatterplot depicting PCA. (**B**) Diagram illustrating the inflection point in PCA. (**C**) Diagram showing the gravel plot corresponding to PCA. (**D**) Dimensionality reduction using Uniform Manifold Approximation and Projection (UMAP). (**E**) Annotation of cell populations. (**F**) Bubble chart displaying marker genes. (**G**) t-Distributed Stochastic Neighbor Embedding (t-SNE) plots representing cellular subtypes. (**H**) Bubble diagrams depicting the expression patterns of hub genes.

Utilizing 2000 highly variable genes post-normalization, no outliers were observed in the PCA scatter plot (Figure 7A). The selection of the initial 17 principal components (PC17) for subsequent analysis was informed by the PCA inflection point diagram and scree plot (Figure 7B,C). Figure 7D depicts the annotation of 12 distinct cell clusters. The identification of six cell types—chondrocytes, dendritic cells (DC), endothelial cells, macrophages, monocytes, and myelocytes—was derived from these annotated cell clusters (Figure 7E). A total of 1466 chondrocytes were isolated by excluding other cell types. Figure 7F–H presents the detailed annotation of chondrocytes based on diverse marker genes (https://doi.org/10.1101/2022.03.25.485846). For example, clusters 2, 6, and 8 were annotated as ProC, and this annotation was based on the expression of FAM101B and LRRC8C (Supplementary Table S3).

### 3.8. Differential Expression of the Two Hub Genes in the Differentiation Stage

**Figure 8:**
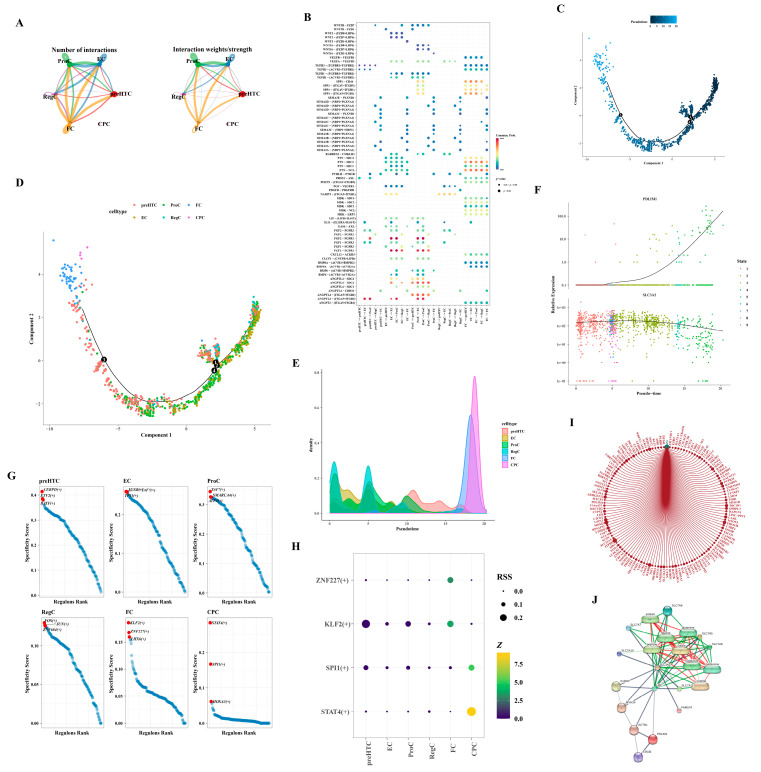
Analysis of intercellular communication. (**A**) Maps illustrating the intercellular communication network. (**B**) Heatmap representing the patterns of intercellular communication. (**C**) Pseudo-temporal trajectory of cells. The black numerated dots denote distinct stages of cell development and differentiation. (**D**) Dendrogram depicting the pseudo-temporal trajectory. The black numerated dots denote distinct stages of cell development and differentiation. (**E**) Density plot showcasing the distribution of the pseudo-temporal trajectory. (**F**) Pseudo-temporal trajectory highlighting the expression patterns of hub genes. (**G**) Transcription factors associated with different subtypes. (**H**) Bubble diagram displaying the transcription factors. (**I**) Network diagram representing the SP4 transcription factor network. (**J**) Network depicting the interactions between hub genes, proteins, and compounds.

Owing to the limited cell count within the CPC subtype, cell communication analysis was exclusively conducted for the remaining subtypes. FC subtype cells exhibited robust interactions with various subtypes, including ProC and FC (Figure 8A,B). Employing the selected feature genes, we constructed a cell pseudo-temporal trajectory (Figure 8C). Notably, the count of RegC and EC subtypes was elevated in the early stages of differentiation, transitioning gradually into ProC and preHTC subtypes, culminating in FC and CPC subtypes by the terminal stages of differentiation (Figure 8D,E). We scrutinized the expression patterns of two hub genes throughout chondrocyte differentiation. *PDLIM1* demonstrated low expression in the early stages, escalating significantly only during the later stages of differentiation. Conversely, *SLC3A2* exhibited relatively high expression throughout, tapering off in the final stages of differentiation (Figure 8F). Figure 8G delineated the TFs associated with the six subtypes, highlighting an enrichment of CEBPD, ETV2, and BATF in the preHTC subtype. In Figure 8H, transcription factors with *Z*-values surpassing three and RSS exceeding 0.1 were presented, with STAT4 (+) being uniquely associated with the CPC subtype. Leveraging the interaction between the SP4 transcription factor and *PDLIM1*, we constructed a network illustrating the interactions of SP4 with target genes in cells. Within this network, SP4 interacted with UTRN, ZNF84, and GCLM (Figure 8I). Based on the database, the network comprised 20 nodes and 84 edges, representing the hub gene–protein/compound interactions. Furthermore, *SLC3A2* demonstrated interactions with various compounds such as L-valine, L-cystine, methionine, isoleucine, glutamine, leucine, arginine, and sodium, as illustrated in Figure 8J.

### 3.9. Expression Levels of SLC3A2 and PDLIM1 in Chondrocytes under Inflammatory Conditions

**Figure 9:**
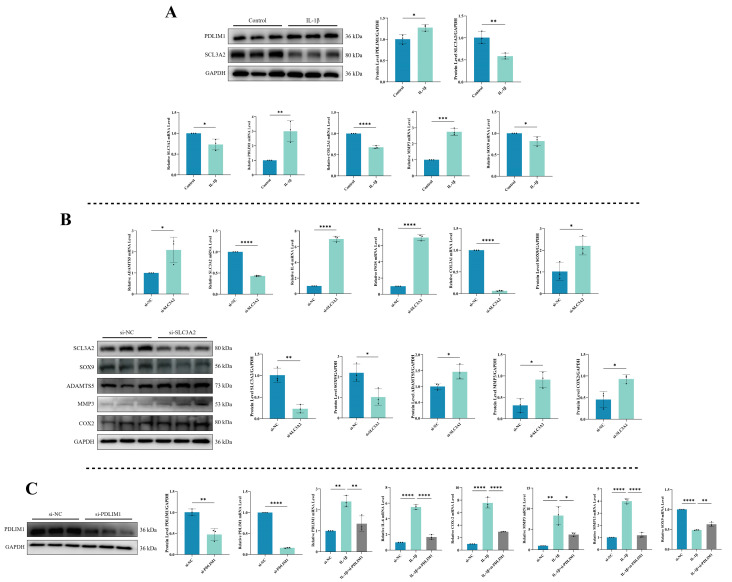
Validation of hub genes. (**A**) Expression levels of *SLC3A2* and *PDLIM1* in chondrocytes under inflammatory conditions. (**B**) Impact of *SLC3A2* knockdown on chondrocytes. (**C**) Consequences of PDLIM1 knockdown in chondrocytes under inflammatory conditions. All experiments and images shown are representative. Each column denotes the mean ± SD derived from three independent experiments. *, *p* < 0.05; **, *p* < 0.01; ***, *p* < 0.001; and ****, *p* < 0.0001.

To understand the expression patterns of DRGs, specifically *SLC3A2* and *PDLIM1*, during inflammation, we utilized a combination of qPCR and Western blot to assess their mRNA and protein levels. Simultaneously, we examined relevant indicators to validate the onset of chondrocytes’ inflammation. As shown in Figure 9A, exposure to IL-1β significantly reduced both the mRNA and protein expression of *SLC3A2* (*p* < 0.05). In contrast, IL-1β stimulation substantially upregulated the mRNA and protein levels of *PDLIM1* (*p* < 0.05). The effectiveness of the chondrocytes’ inflammation model was unequivocally substantiated by the significant increase in *MMP3* expression following IL-1β exposure, while *COL2A1* and *SOX9* levels were found to be downregulated (*p* < 0.05).

### 3.10. Impact of SLC3A2 Knockdown on Chondrocytes

To explore *SLC3A2*’s role in chondrocytes, we conducted a knockdown experiment to assess its effects (Figure 9B). *SLC3A2* knockdown led to an increase in the mRNA expression of cartilage catabolic markers and inflammatory factors (*ADAMTS5*, *IL-6*, and *iNOS*) (*p* < 0.05), accompanied by decreased *SLC3A2* and mRNA expression of cartilage anabolic markers (*COL2A1* and *SOX9*) (*p* < 0.05). Additionally, there was an increase in the protein expression of cartilage catabolic markers, including *ADAMTS5*, *MMP3*, and *COX-2* (*p* < 0.05), alongside a decrease in *SOX9* protein expression (*p* < 0.05). These findings suggest that the reduction in *SLC3A2* expression may play a crucial role in triggering inflammation in chondrocytes.

### 3.11. Consequences of PDLIM1 Knockdown in Chondrocytes under Inflammatory Conditions

We evaluated the effectiveness of *PDLIM1* knockdown using siRNA through qPCR and Western blot to measure *PDLIM1* expression (Figure 9C). The outcomes showed a significant decrease in both *PDLIM1* mRNA and protein levels (*p* < 0.05). To further confirm *PDLIM1*’s role in chondrocytes under inflammatory conditions, we exposed chondrocytes to IL-1β for 24 h, followed by si-PDLIM1 knockdown, and conducted qPCR experiments for validation. The results revealed that treating chondrocytes in an inflammatory state with si-PDLIM1 led to a reduction in *IL-6*, *COX-2*, *MMP3*, and *MMP13* levels, along with a restoration of *SOX9* expression (*p* < 0.05).

## 4. Discussion

OA, a chronic orthopedic degenerative condition, involves cartilage deterioration, osteophyte formation, and joint cavity inflammation, significantly impacting the quality of life, especially in the elderly [36,37]. Disulfidptosis, a novel type of PCD, arises from disulfide accumulation within cells, leading to abnormal disulfide bond formation in cytoskeletal actin and subsequent F-actin collapse [18]. Disulfidptosis plays a crucial role in the pathogenesis of various diseases. In this study, we utilized single-cell and transcriptomic data to identify hub genes associated with disulfidptosis in OA. Additionally, we explored potential regulatory mechanisms of DRGs to offer new insights for OA diagnosis and management.

### 4.1. Implication of Hub Genes in the Biological Processes of OA

In the present study, we identified a set of key genes, collectively called DE-DRGs, which includes *SLC3A2*, *INF2*, *PDLIM1*, *MYL6*, *ACTB*, and *CAPZB*. We applied three different machine learning techniques, specifically RF, Treebag, and elastic net, to build a predictive model. In this model, we highlighted *SLC3A2* and *PDLIM1* as crucial genes with high diagnostic potential (AUC > 0.8). Notably, *SLC3A2* was found to be downregulated in patients with OA, while *PDLIM1* showed an upregulation. Our analysis using KEGG revealed that genes positively correlated with *SLC3A2* were mainly associated with autophagy, a process vital for the breakdown and clearance of damaged cell components, often linked to various aging-related conditions [38]. Considering that OA is a degenerative condition often associated with aging, it leads to a reduction in cartilage autophagy levels. However, activating autophagy can help mitigate chondrocyte apoptosis and protect them from degradation [39,40,41]. On the other hand, our GO analysis demonstrated that genes positively linked with *PDLIM1* were prominently associated with the extracellular matrix, particularly collagen. Collagen, serving as a primary component around chondrocytes, becomes a significant target for cartilage degradation in OA [42]. We also conducted a detailed analysis of single-cell data, focusing on 2000 highly variable genes from a pool of 1471 cells (Figure 6A–F). Among these, we identified ten highly variable genes of interest: *IBSP* (related to cartilage calcification), *COL1A1* (associated with cartilage dedifferentiation), *PRG4* (involved in cartilage anabolism), HSPA6 (linked to endoplasmic reticulum stress), *HSPA7*, *KRT17*, *COL10A1* (related to chondrocyte hypertrophy), *CCL2* (a chemokine associated with pain), *TAC* (linked to antioxidant capacity), and *ILRL1* (an interleukin receptor). A subgroup analysis of chondrocytes revealed that RegC and EC subtypes mainly represented early differentiation stages, while ProC, preHTC, FC, and CPC subtypes emerged as dominant subtypes in later stages of differentiation (Figure 8D,E). Notably, *PDLIM1* exhibited a significant surge in expression during the late differentiation stage, while *SLC3A2* displayed a declining trend at the same stage (Figure 8F). Therefore, we hypothesize that the expression levels of *SLC3A2* and *PDLIM1*, recognized as genes associated with disulfidptosis, may correlate with OA severity and could serve as markers for detecting late-stage chondrocyte differentiation. Further study is warranted to validate this discovery and gain a deeper understanding of the underlying mechanism.

### 4.2. Involvement of SLC3A2 and PDLIM1 in the Pathogenesis of OA as Hub Genes

*SLC3A2*, also known as CD98, is a transmembrane glycoprotein that forms a vital partnership with *SLC7A11* to facilitate amino acid transport through System Xc^-^ [43]. While *SLC3A2*’s connection to disulfidptosis is not extensively explored, it deserves attention as a significant DRG. Jiang et al. presented compelling evidence supporting *SLC3A2*’s potential as a diagnostic and therapeutic marker in bladder cancer, linked to disulfidptosis [44]. Ni et al. also suggested its relevance to the survival rates of patients with lung adenocarcinoma, designating it as a promising prognostic biomarker [45]. In the context of OA, Liu et al. conducted bioinformatics analysis coupled with in vitro experiments, revealing a notable downregulation of *SLC3A2* expression in OA [46]. In our validation experiments, IL-1β treatment or siRNA-induced *SLC3A2* silencing led to an increased mRNA expression of cartilage catabolic markers and inflammatory factors, along with a decrease in mRNA expression related to cartilage anabolic markers. Importantly, these trends were reflected at the protein level, reinforcing our conclusion that *SLC3A2*, identified as a hub gene associated with disulfidptosis, might contribute to driving OA progression when its expression is diminished. These findings underscore the potential significance of *SLC3A2* in the pathogenesis of OA and advocate for further investigation into its mechanisms and therapeutic implications. Subsequent studies could delve into the specific role of *SLC3A2* in regulating amino acid transport and its impact on chondrocyte homeostasis. Moreover, exploring *SLC3A2* expression as a potential modulator of therapeutic strategies for OA could offer valuable insights into disease management.

Another notable protein, *PDLIM1* (also known as *CLP36*, *Elfin*, or *CLIM1*), is a conserved regulator of cytoskeletal organization, contributing to cellular homeostasis [47]. It primarily colocalizes with α-actinins and actin stress fibers across various cell types, potentially influencing the functionality of α-actinin-1 [48]. Joos et al. investigated the impact of the proinflammatory cytokine IL-1β on the cytoskeletal components of chondrocytes in OA. They found a significant reduction in *PDLIM1* mRNA expression levels within 24 h of IL-1β exposure, particularly in primary chondrocytes from osteoarthritic cartilage in individuals aged 58 to 72 years [49]. Building on this, they proposed a hypothesis suggesting that reduced *PDLIM1* presence inhibits actin polymerization and local complex assembly in chondrocytes, influencing chondrocyte motility, migration capacity, and the reparative potential of cartilage.

Chen et al. used an adenovirus system to inject mice at the fracture site, finding that reducing *PDLIM1* led to increased RUNX2 expression and activated β-catenin, ultimately encouraging early cartilage callus formation [50]. Both RUNX2 and β-catenin are crucial factors in the onset of OA [51]. In contrast, Ye et al., studying *PDLIM1*’s impact on hepatic stellate cell activation and liver fibrosis progression, observed reduced levels of inflammatory factors (*TNF-α*, *IL-6*, and *p65*) after *PDLIM1* knockdown [52]. Helga Joos et al. noted a decrease in *PDLIM1* mRNA expression in human primary chondrocytes treated with IL-1β. However, our experiments showed increased *PDLIM1* mRNA and protein expression in mouse chondrocytes after IL-1β exposure. Additionally, si-PDLIM1 knockdown in IL-1β-treated chondrocytes reduced the mRNA expression of inflammatory factors and cartilage catabolic markers (*IL-6*, *COX-2*, *MMP3*, and *MMP13*). These differences may arise from variations in differentiation status between human primary chondrocytes and primary mouse chondrocytes, along with their distinct responses to IL-1β.

In our single-cell analysis, we conducted cell communication analysis and traced pseudo-time for categorized chondrocyte subtypes. Initially, *PDLIM1* expression was low in early chondrocyte differentiation. However, as chondrocytes progressed into late stages, differentiating into ProC, preHTC, FC, and CPC subtypes, there was a significant and rapid increase in *PDLIM1* expression during late differentiation (Figure 8F). We propose that *PDLIM1* expression is upregulated during chondrocyte differentiation from early to late stages in our IL-1β-induced chondrocyte inflammation in vitro model. Interestingly, in primary human OA chondrocytes at the late differentiation stage, we observed a downregulation of the cytoskeleton-related gene *PDLIM1* due to interference from inflammatory factors. Zhang and Shang et al.’s study revealed that *PDLIM1* accumulation inhibits autophagy, crucial for degrading negative regulators in cytoskeleton organization. Autophagy maintains the normal cytoskeleton morphology, including F-actin and microtubule organization, preventing cytoskeleton collapse [53,54]. In OA, *PDLIM1* accumulates in chondrocytes during the late differentiation stage, inhibiting autophagy. Furthermore, *PDLIM1* competitively binds to the cytoskeletal cross-linking protein α-actin 4 (ACTN4), causing ACTN4 to dissociate from F-actin [55]. This dissociation might contribute to the collapse of F-actin in the cytoskeleton and, consequently, disulfidptosis [14].

Our bioinformatics and single-cell sequencing analyses suggest that *SLC3A2* and *PDLIM1* can serve as indicators for tracking chondrocyte differentiation stages and detecting OA. These markers also provide insights into the role of disulfidptosis in OA progression. Notably, this study is the first to link disulfidptosis with OA, offering new insights into the molecular mechanisms of OA. However, it is important to note certain limitations in our study. Firstly, the inherent heterogeneity among the datasets utilized in the present study might introduce bias and influence the robustness of the findings. Secondly, the absence of experiments involving both animal and human tissue samples inevitably affects the universality and applicability of our findings. Therefore, future exploration through prospective studies and experimental research using animal models and clinical OA samples is essential to refine the details of the present study. The authors will continue to work on the framework of the project described above.

## 5. Conclusions

In this study, we first combined single-cell and transcriptome data to investigate the connection between DRGs and OA. We then explored the involvement of *SLC3A2* and *PDLIM1* in inflamed chondrocytes, indicating that disulfidptosis plays a role in OA progression. The altered expression of *SLC3A2* and *PDLIM1* could serve as indicators of OA severity, highlighting their biological significance. These findings lay the groundwork for developing novel targeted therapies and clinical diagnostic tools for managing OA.

## Data Availability

Researchers seeking access to the datasets utilized or analyzed in this study may submit a reasonable request to the corresponding authors.

## Supplementary Materials

The supporting information can be downloaded at: https://www.mdpi.com/article/10.3390/biomedicines12081840/s1. Table S1. The Specific Sequences of siRNAs targeting *SLC3A2* and *PDLIM1*. Table S2. The Primer Sequences for qPCR. Table S3. Annotation of Six Cell Types from Twelve Cell Clusters.
