# Supplementary material for: Bioinformatics Identification and Experimental Verification of Disulfidptosis-Related Genes in the Progression of Osteoarthritis
Source: Biomedicines. 2024 Aug 13;12(8):1840. doi: 10.3390/biomedicines12081840 (PMC11351109; doi:10.3390/biomedicines12081840)
Supplement: Supplementary file 1 [file biomedicines-12-01840-s001.zip › biomedicines-3094195-supplementary.pdf]

Supplementary Material

Bioinformatics Identification and Experimental Verification of Disulfidptosis-Related Genes in the Progression of Osteoarthritis

Siyang Cao [1,2,3,†], Yihao Wei [1,2,3,†], Yaohang Yue [1,2,3], Deli Wang [1,2,3], Ao Xiong [1,2,3,*], Jun Yang [4] and Hui Zeng [1,2,3,*]

[1] National & Local Joint Engineering Research Centre of Orthopaedic Biomaterials, Peking University Shenzhen Hospital, Shenzhen 518036, China
[2] Shenzhen Key Laboratory of Orthopaedic Diseases and Biomaterials Research, Peking University Shenzhen Hospital, Shenzhen 518036, China
[3] Department of Bone & Joint Surgery, Peking University Shenzhen Hospital, Shenzhen 518036, China
[4] Department of Radiology, Peking University Shenzhen Hospital, Shenzhen 518036, China
* Correspondence: xiongao@189.cn (A.X.); zenghui@pkuszh.com (H.Z.)
† These authors contributed equally to this work and share the first authorship.

Supplementary Tables

Supplementary Table S1. The Specific Sequences of siRNAs targeting *SLC3A2* and *PDLIM1*.

**Type of siRNA**	**Sequence**
***SLC3A2* siRNA**	
No. 1	
Sense	5’-GGAGCUGAACGAGCUAGAATT-3’
Antisense	5’-UUCUAGCUCGUUCAGCUCCTT-3’
No. 2	
Sense	5’-GGGUCAAGUUCACCGGCUUTT-3’
Antisense	5’-AAGCCGGUGAACUUGACCCTT-3’
No. 3	
Sense	5’-GGAAUGAGUCCAGCAUCUUTT-3’
Antisense	5’-AAGAUGCUGGACUCAUUCCTT-3’
***PDLIM1* siRNA**	
No. 1	
Sense	5'-GCCAUUUACUGCCUCACCUTT-3’
Antisense	5'-AGGUGAGGCAGUAAAUGGCTT-3’
No. 2	
Sense	5'-GAUGAAUUUAGCGUCGGAATT-3’
Antisense	5'-UUCCGACGCUAAAUUCAUCTT-3’
No. 3	
Sense	5'-GCUGCUAUAGCGAAUUUAUTT-3’
Antisense	5'-AUAAAUUCGCUAUAGCAGCTT-3’
No. 4	
Sense	5'-CCAGCAGCAUGACACACUUTT-3’
Antisense	5'-AAGUGUGUCAUGCUGCUGGTT-3’

Supplementary Table S2. The Primer Sequences for qPCR.

Primers	Sequences
GAPDH	
Forward	5’-AGGTCGGTGTGAACGGATTTG-3’
Reverse	5’-TGTAGACCATGTAGTTGAGGTCA-3’
MMP3	
Forward	5’-ACATGGAGACTTTGTCCCTTTTG-3’
Reverse	5’-TTGGCTGAGTGGTAGAGTCCC-3’
COL2A1	
Forward	5’-GGGAATGTCCTCTGCGATGAC-3’
Reverse	5’-GAAGGGGATCTCGGGGTTG-3’
SOX9	
Forward	5’-GAGCCGGATCTGAAGAGGGA-3’
Reverse	5’-GCTTGACGTGTGGCTTGTTC-3’
PDLIM1	
Forward	5’-TCGATGGGGAAGATACCAGCA-3’
Reverse	5’-TCTGTTCAGACCTGGATACTGTG-3’
SLC3A2	
Forward	5’-TGATGAATGCACCCTTGTACTTG-3’
Reverse	5’-GCTCCCCAGTGAAAGTGGA-3’
IL-6	
Forward	5’-TAGTCCTTCCTACCCCAATTTCC-3’
Reverse	5’-TTGGTCCTTAGCCACTCCTTC-3’
ADAMTS5	
Forward	5’-GGAGCGAGGCCATTTACAAC-3’
Reverse	5’-CGTAGACAAGGTAGCCCACTTT-3’
COX-2	
Forward	5’-TTCAACACACTCTATCACTGGC-3’

Reverse	5'-AGAAGCGTTTGCGGTACTCAT-3'
MMP13	
Forward	5'-CTTCTTCTTGTTGAGCTGGACTC-3'
Reverse	5'-CTGTGGAGGTCACTGTAGACT-3'
iNOS	
Forward	5'-GTTCTCAGCCCAACAATACAAGA-3'
Reverse	5'-GTGGACGGGTCGATGTCAC-3'

Supplementary Table S3. Annotation of Six Cell Types from Twelve Cell Clusters.

Cluster	Marker	Subtype
2, 6 and 8	FAM101B and LRRC8C	ProC
1	CHRDL2 and DSC2	EC
0, 5 and 10	TGFB1, CRLF1, MXRA5 and THBS3	preHTC
7	TMEM176A	RegC
3	SNHG12, KLF2, MYLIP and RPPH1	HomeC
4	BHLHE41 and CRISPLD1	HTC
11	STMN1, KCNN4, and C7orf73	CPC
9	IFI27, COL1A1, THY1 and COL14A1	FC
